# Nanostructured Copper Selenide Coatings for Antifouling Applications

**DOI:** 10.3390/polym16040489

**Published:** 2024-02-09

**Authors:** Sergio Mancillas-Salas, José Ángel Ledón-Smith, Marissa Pérez-Álvarez, Gregorio Cadenas-Pliego, José Manuel Mata-Padilla, Marlene Andrade-Guel, Sandra Cecilia Esparza-González, Gregorio Vargas-Gutiérrez, Uriel Alejandro Sierra-Gómez, Esmeralda Monserrat Saucedo-Salazar

**Affiliations:** 1 Centro de Investigación en Química Aplicada, Blvd. Enrique Reyna 140, Saltillo 25294, Coahuila, Mexico; sergio.mancilla.ps@ciqa.edu.mx (S.M.-S.); jangel.ledon.m20@ciqa.edu.mx (J.Á.L.-S.); marlene.andrade@ciqa.edu.mx (M.A.-G.); uriel.sierra@ciqa.edu.mx (U.A.S.-G.); esmeralda.saucedo@ciqa.edu.mx (E.M.S.-S.); 2 CONAHCYT, Centro de Investigación en Química Aplicada, Blvd. Enrique Reyna 140, Saltillo 25294, Coahuila, Mexico; jose.mata@ciqa.edu.mx; 3 Facultad de Odontología, Unidad Saltillo, Universidad Autónoma de Coahuila, Saltillo 25125, Mexico; sceciliaesparza@gmail.com; 4 Centro de Investigación y de Estudios Avanzados del IPN, CINVESTAV Unidad Saltillo, Ramos Arizpe 25900, Coahuila, Mexico; gregorio.vargas@cinvestav.edu.mx

**Keywords:** copper selenide, antimicrobial activity, nanoparticles synthesis, nanostructured coating

## Abstract

The accumulation of microorganisms, plants, algae, or small animals on wet surfaces that have a mechanical function causes biofouling, which can result in structural or other functional deficiencies. The maritime shipping industry must constantly manage biofouling to optimize operational performance, which is a common and long-lasting problem. It can occur on any metal structure in contact with or submerged in ocean water, which represents additional costs in terms of repairs and maintenance. This study is focused on the production of antifouling coatings, made with nanoparticles of copper selenide (CuSe NPs) modified with gum arabic, within a water-base acrylic polymeric matrix. During the curing of the acrylic resin, the CuSe NPs remain embedded in the resin, but this does not prevent the release of ions. The coatings released copper and selenium ions for up to 80 days, and selenium was the element that was released the most. The adhesion of film coatings to metallic substrates showed good adhesion, scale 5B (ASTM D3359 standard). Antimicrobial activity tests show that the coatings have an inhibitory effect on *Escherichia coli* and *Candida albicans*. The effect is more noticeable when the coating is detached from the substrate and placed on a growing medium, compared to the coating on a substrate. Scanning electron microscopy (SEM) observations show that nanostructured CuSe coatings are made up of rod-shaped and spherical particles with an average particle size of 101.6 nm and 50 nm, respectively. The energy dispersive X-ray spectroscopy (EDS) studies showed that the ratio of selenium nanoparticles is greater than that of copper and that their distribution is homogeneous.

## 1. Introduction

Biofouling can be defined as the adhesion of micro- and macroorganisms to a metal structure submerged in ocean waters, and more than 4000 species have been identified in this process [1]. Its main consequence is the deterioration of the affected metals, derived from the corrosion induced by microorganisms (CIM), a fact that causes damage and weakening of the structures, increasing maintenance costs and their periodicity [2]. It is estimated that the damage associated with corrosion per year ranges from 30 to 50 billion US dollars [3].

Biofouling is also associated with the increase in the use of fuel from ships and its consequent increase in environmental pollution; due to the increase in friction during their displacement, economic studies have established that biofouling can increase fuel consumption between 40 and 77% [3]. An analysis of the economic impact of biofouling in the US Navy fleet indicates an additional cost of between USD 180 and 260 million per year [4]. The advantages of using antifouling coatings on yachts have been evaluated, taking into account the reduction in fuel consumption and the corresponding reduction in CO_2_ emissions, and it was found that the fuel reduction in one year can be approximately 13.7 × 10^3^ kg and CO_2_ emissions can be reduced by 43.3 tons [5].

Other damages caused by biofouling include the clogging of sewage pipes and biofouling overgrowth on offshore oil or power generation platforms, which become heavier and less resistant to marine wear [6]. Another important consequence of the formation of the biofouling would be the ecological alteration caused by the artificial introduction of organisms (through dragging) from one ecosystem to another [4,7].

The prevention of biofouling phenomena is a widespread issue dealing with a plethora of research fields focused on the design of highly performing nanocomposite materials. Research on antifouling coatings involves water purification systems, marine equipment, biomedical devices, food packaging, fabrics, and heritage materials [1,8,9].

The increasing interest in the development of antifouling coatings has led to the development of different types of coatings that are used today. Decades ago, coatings made with paints embedded in cytotoxic agents were freely employed, whose function was the gradual release of these agents into the environment, but their use was restricted/limited due to the environmental deterioration they caused [10]. Coatings that facilitate removal (foul-release) have a hydrophobic surface where aquatic organisms have low adherence, which facilitates their removal. Some of these coatings can be gradually hydrolyzed, releasing their surface layer, thus limiting biofouling [11,12]. Finally, another type of antifouling coatings are those that usually employ a polymer matrix endowed with nanoparticles, which in combination have an effect that limits or inhibits microbial growth [13]. In addition, the surface roughness characteristics of these coatings are also a limitation for biofouling [14].

The type of nanoparticles and their interaction with the polymer matrix are the main factors to consider in the development of antifouling coatings. The use of copper as an antimicrobial agent has been known since antiquity and its use as nanoparticles is well documented [15,16]. In the last decade, the interest in synthesizing copper nanoparticles [17,18] and copper nanocomposites has increased significantly [19,20], since their excellent properties allow them to have applications in different fields of science.

Selenium is an element with biological importance, since it forms part of most living beings, it has low toxicity and excellent antimicrobial properties, and it is used in various products related to the safety of human health, for example, in the generation and handling of food [8], as an antioxidant [21,22], for its anti-carcinogenic properties [22], and therapeutical applications [23], among others.

Copper selenide (CuSe), on the other hand, is a chalcogenide commonly used in applications such as photovoltaics, thermoelectrics, and electronics [24,25,26]. It is a compound capable of being presented in different compositions and very complicated crystalline structures [25,26,27,28]. The Cu/Se ratio plays an important role in the properties of the material, and it was reported that hexagonal α-CuSe formed with 60% selenium is converted to hexagonal γ-CuSe at ~130 °C [28]. In the case of selenium NPs, the transformation can occur at lower temperatures [25].

Coatings with metal nanoparticles such as Ag and Cu have been proven to have excellent antibacterial properties; however, this property can be inefficient over time because bacteria can develop resistance to the nanoparticles. In the last decade, superhydrophobic (self-cleaning) coatings have emerged as a durable solution to control the spread of bacteria, because they are purely structural and do not develop bacterial resistance. Recent research combines the antimicrobial effects of superhydrophobic coatings and coatings with biocides such as Cu, Ag, Zn, or TiO_2_ [29,30]. Superhydrophobic coatings have a physical antibacterial action, they prevent the adhesion of bacteria because they have contact angles greater than 150°, and they are considered environmentally friendly and long-lasting; this last property has recently been controversial [30,31].

Little has been said about its antimicrobial properties, but considering that copper and selenium present excellent antimicrobial properties separately, CuSe may be a promising candidate in this field.

In the present work, we developed nanostructured antifouling coatings that consist of copper selenide (CuSe) nanoparticles and a commercial acrylic resin named Rhoplex Ac-261 and their surface, mechanical, and antimicrobial properties were evaluated.

## 2. Materials and Methods

The synthesis of CuSe NPs was carried out using copper (II) sulfate pentahydrate (CuSO_4_·5H_2_O) ACS reagent ≥ 98%, gun arabic ≥ 97%, selenous acid (H_2_SeO_3_) ACS reagent 97% and hydrazine hydrate (N_2_H_4_·H_2_O, reagent grade, 50–60%). All chemicals were supplied by Sigma Aldrich (St. Louis, MO, USA) and were used as received without further treatment. For the preparation of the CuSe nanostructured coatings, CuSe NPs and an acrylic resin with the trade name Rhoplex Ac-261 (Dow Chemical Inc., Midland, MI, USA) were used.

### 2.1. CuSe Nanoparticle Synthesis

The chemical synthesis of CuSe nanoparticles was carried out in our work group using a similar methodology to that previously reported [21]. In a 2 L capacity glass reactor, 1.25 L of distilled water and 3.0 g of gum arabic (GA) were introduced and stirred at 200 rpm for 15 min. Then, 5.2 g of H_2_SeO_3_ was added to the mixture and the stirring was incremented to 330 revolutions per minute (rpm) to ensure that the reaction was homogeneous; stirring was maintained for 50 min. Then, a solution of CuSO_4_·5H_2_O (0.16 M) was added to the reaction mixture. At this point, the reactor was hermetically closed, and the stirring was incremented to 400 rpm. After, 9 mL of hydrazine (N_2_H_4_) was added dropwise and the reactor stirring was maintained for another 60 min. The mixture reaction solution was separated by centrifugation at 8500 rpm for 30 min at room temperature. The collected black solid products were washed twice with distilled water and ethanol and dried under vacuum at 60 °C for 1 h, the average particle diameter was 7.0 nm ± 0.5. The molar ratio Se/Cu used in this project was 1.24.

### 2.2. Preparation of CuSe Nanostructured Coatings and Deposition

Stainless steel 304 (SS–304) substrates were used for the preparation of the nanostructured coatings. The substrates were first treated to eliminate oxides, deformations, and defects by surface shot blasting following the ASTM-A380/A380M-13 [32]. First, abrasive papers of the following grain sizes were used: 280, 400, 600, 800, and 1000. They were used from the largest grain size to the smallest, to ensure deep cleaning, necessary for the correct adhesion of the nanostructured coatings; they abraded both the faces and corners of the substrates; and the process was carried out wet, using deionized water. After polishing, the samples were washed with deionized water and placed in a 10% NaOH solution for 7 min, to remove fats and contaminants that were on its surface and that prevent the coating from adhering correctly. They were washed first with plenty of deionized water and finally with ethanol, and then they were allowed to air dry. The treated substrates were used less than 24 h after drying, to avoid the formation of oxides.

The preparation of the formulations was performed in two steps. First, it consisted of suspending the nanoparticles in water; due to the nature of the nanoparticles, this step required the use of ultrasound equipment. The conditions were established at 70 kHz for a time of 15 min, and after, the solution was cooled to room temperature and the dispersion was verified by measuring the zeta potential. The second step consisted of adding the solution at room temperature to the resin to form the nanostructured coating. For this purpose, it was necessary to mix it in a reactor (Parr, 4842) at 400 rpm for 10 min to ensure correct mixing.

The coating was deposited using the doctor blade technique, the coated substrates were kept at room temperature for 30 min, and then they were subjected to heat treatment, using temperatures of 80 °C and 130 °C for 1 h. Thermal curing was carried out in an oven, in daylight and air.

Optical microscopy was used to analyze the coatings and observe the microstructure of the nanoparticles; for this, an optical microscope with a 4× magnification lens was employed.

Fourier transformed infrared spectroscopy (FTIR) was performed with a Thermo Scientific Nicolet iS20 using an ATR.

A scanning electron microscope (SEM) was used to analyze the microstructure, and the samples were prepared by applying a coating formulation composed of commercial resin, water, and 3.0% by weight of CuSe nanoparticles on a 1 mm × 1 mm stainless steel substrate. The sample was coated with gold and palladium before analysis. The technique was carried out in a JEOL JSM-7001F electron microscope (Jeol LTD., Akishima, Tokyo, Japan) operated at 8 kV. 

For ion release analysis, the samples were placed in a flask with 20 mL of distilled water. The flask was closed, and the nanostructured coatings were kept at rest for 5, 10, 20, 40, and 80 days. Subsequently, the water of all flasks was analyzed by inductively coupled plasma spectrometer iCAP 7000 series model (Thermo Scientific Inc., Waltham, MA, USA). Distilled water was used as a blank. The swollen samples, at 5, 10, 20, 40, and 80 days, were dried at room temperature. These samples underwent a surface roughness analysis, and the contact angle was measured.

The coatings’ roughness was measured by a Keyence VR Series profilometer. Contact angle measurements were performed by a Rame-Hart goniometer using 3 µL of distilled water in each measurement.

### 2.3. Mechanical Performance Tests

The adhesion of CuSe nanostructured coatings to the stainless steel substrate was determined based on the ASTM D3359 standard (American Standard Test Methods for Measuring Adhesion by Tape Test) [33]. As established by the test, a lattice pattern with eight cuts is made in each direction through the film to the substrate, pressure-sensitive tape is applied to the web pattern and then removed, and qualitative adhesion is assessed on a scale of 0 to 5 B.

### 2.4. Microbiological Test

For the antimicrobial tests, strains of *Escherichia coli* (Gram-negative bacteria) and *Candida albicans* (fungi) were used. Nutrient agar (BD Bioxon) was employed and prepared according to the product instructions. Subsequently, the culture medium was sterilized in an autoclave at a temperature of 121 °C and 15 psi of pressure for 15 min. Finally, it was cooled at room temperature.

CuSe nanostructured coatings were prepared at concentrations of 0, 0.5, 1.0, 1.5, 2.0, and 3.0% weight of CuSe nanoparticles (2 for each concentration), and the culture medium was applied directly to the coatings. Subsequently, on the surface of each agar, a perforation was made where 10 µL of inoculum was poured (each inoculum for all 6 concentrations). Finally, these samples were incubated for 24 h.

To validate these results, a special test was carried out, using only the coating without the substrate (a thin film). New culture mediums were prepared and inoculated with both microorganisms, then the thin film was placed on the inoculated agar. The concentrations used were the same as before, obtaining 12 treatments. Similarly, the samples were incubated for 24 h and then observed in an optical microscope at 40×.

The inoculums or strains were prepared in tubes containing nutrient broth and incubated at 37 °C for 24 h until the medium had a turbidity of 1 McFarland. For this technique, the inoculum (1 on the McFarlad scale) was prepared with different microorganisms such as C. albicans (Sabouraud dextrose broth) and E. coli (nutrient broth). Sowing was carried out on each of the surfaces of the plates with Sabouraud agar and nutrient agar using a swab with the suspensions of the microorganisms; the cut 1 cm × 1 cm films of each of the different concentrations placed on the surface of the agar were brought into contact, previously inoculated. Subsequently, the plates were incubated at 37 °C for 24 h for bacteria; and for fungi, they were incubated at 25 °C for 24 h. Subsequently, the images were taken using a 40× microscope with a Dinolite camera.

The samples were cleaned with 70% ethanol and washed with water to be placed in sterile Petri dishes. The samples were inoculated with 0.05 mL covering the entire surface and subsequently incubated under the conditions described for 24 h. Next, 10 mL of sterile 1× PBS was added to sonicate them. The solutions obtained from each sample were subjected to serial dilutions to later be sown on agar and incubated to determine the number of colony-forming units [34].

## 3. Results and Discussion

### 3.1. CuSe Nanostructured Coatings’ Characterization

**Figure 1:**
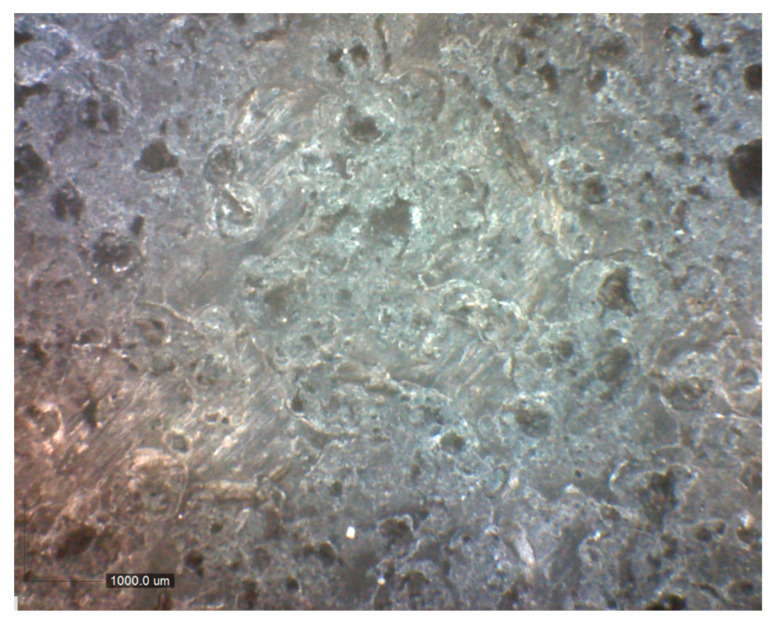
Optical image of CuSe nanostructured coating at 4×.

Figure 1 presents the optical image of CuSe antifouling coating at 4×. It can be appreciated that this coating presents a not-homogeneous distribution due to the formation of bubbles during the drying and curing of the coating. Imperfections on the surface can be observed even when different layers of the coating are applied.

**Figure 2:**
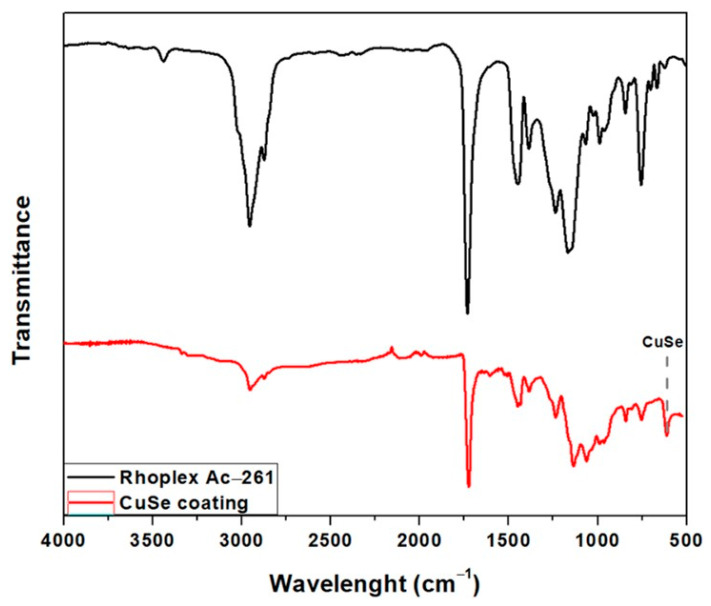
FTIR spectra of CuSe nanostructured coating and Rhoplex Ac-261 commercial resin.

Figure 2 presents the FTIR spectra of the CuSe nanostructured coating and the Rhoplex Ac-261 commercial resin as a blank. The Rhoplex Ac-261 acrylic resin spectrum presents bands between 3000 and 2800 cm^−1^, 1730 cm^−1^, and 1030 and 1250 cm^−1^, which can be assigned to bands of C-H bonds, carbonyl groups, and C-O, respectively. The detailed analysis of the spectrum shows characteristic signals at 2958 cm^−1^, 2882 cm^−1^, 1730 cm^−1^, 1448 cm^−1^, and 1152 cm^−1^, where the first two are attributed to the stretching of the -CH group, the third one belongs to the stretching of the C=O group, the fourth signal is due to the stretching of the -CH_3_ group, and the last one is due to the stretching and vibrations of O-CH_3_ corresponding to the ester group. The presence of the band at 3446 cm^−1^ suggests the presence of hydroxyl groups (-OH) or water molecules trapped in the test material [35]. The bands described are characteristic of polymethylmethacrylate (PMMA); however, the absorption band at 961 cm^−1^ is typical of polybutylacrylate (PBA), and this band is due to the oscillation of the carbon of the -COO group of the PBA, as found in the literature [36]. PMMA and PBA had similar molecular backbones; only slight differences were found in their FTIR spectra, and the presence of these segments suggests the presence of the copolymer P(MMA-co-BA) [37].

The spectrum of the nanostructured coating with CuSe nanoparticles turned out to be very similar; the main difference found is the peak localized at 611 cm^−1^, which is attributed to the bending vibrations of CuSe [26]. Modifications and shifts in the nanostructured CuSe coating spectrum compared to the Rhoplex Ac-261 spectrum are attributed to an interaction with CuSe-GA (see Section 2.1) nanoparticles. As reported, predominant groups of gum arabic can be shown in the spectrum of the modified nanoparticles [38].

**Figure 3:**
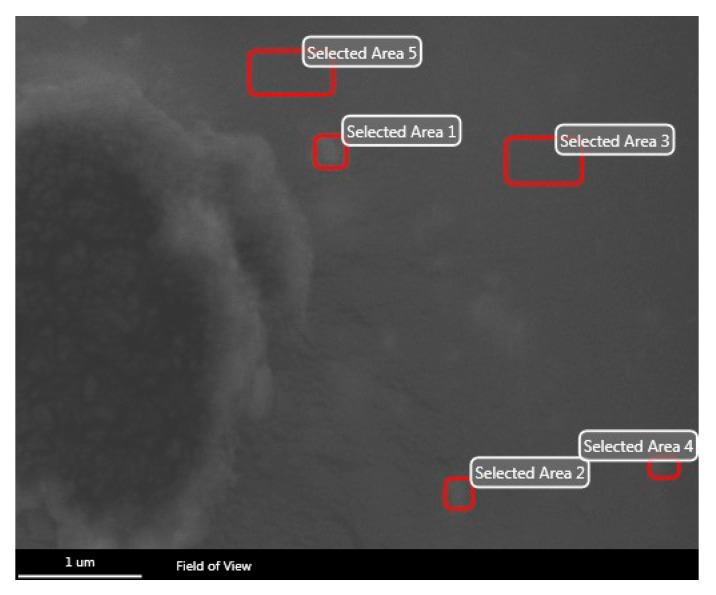
SEM micrograph of the CuSe nanostructured coating for elemental analysis.

Figure 3 shows the SEM micrograph of the CuSe nanostructured coating for elemental analysis. In this analysis, five zones were selected to carry out the elemental analysis, and zones 1, 2, and 3 were the ones that presented a greater number of particles compared to zones 4 and 5.

According to the analysis, the presence of copper and selenium was observed in all areas. This demonstrates that the copper and selenium particles are embedded in the resin and that the dispersion and depth of the particles are varied. 

**Figure 4:**
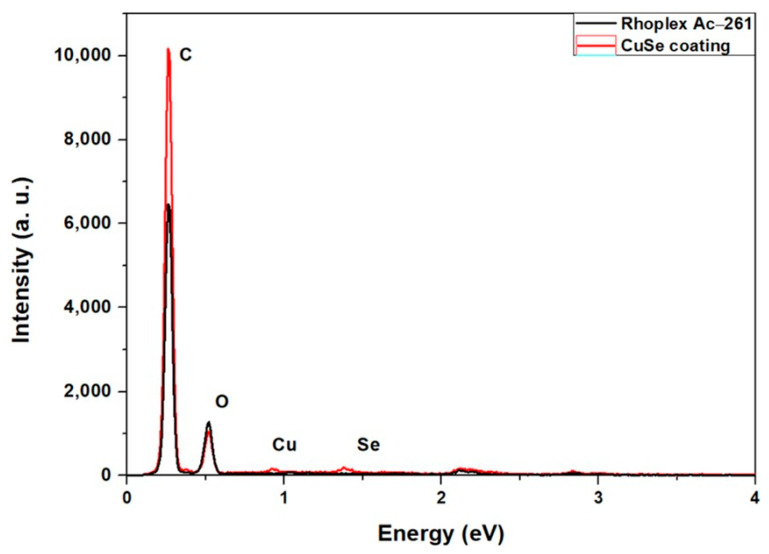
EDAX analysis of the CuSe nanostructured coating.

Figure 4 shows a comparison of the spectra of the Rhoplex Ac-261 commercial resin and the CuSe coating obtained from the elemental analysis (EDS), where the signals that are not labeled correspond to the elements gold and palladium used in the preparation of the sample.

Table 1 shows the concentration in weight percentage of the elements carbon, oxygen, selenium, and copper. The average of the first three zones showed that the amount of selenium in the coating was greater than the amount of copper (Se/Cu = 1.35); on the other hand, the carbon and oxygen elements are derived from the chemical structure and functional groups of resin and gum arabic.

**Figure 5:**
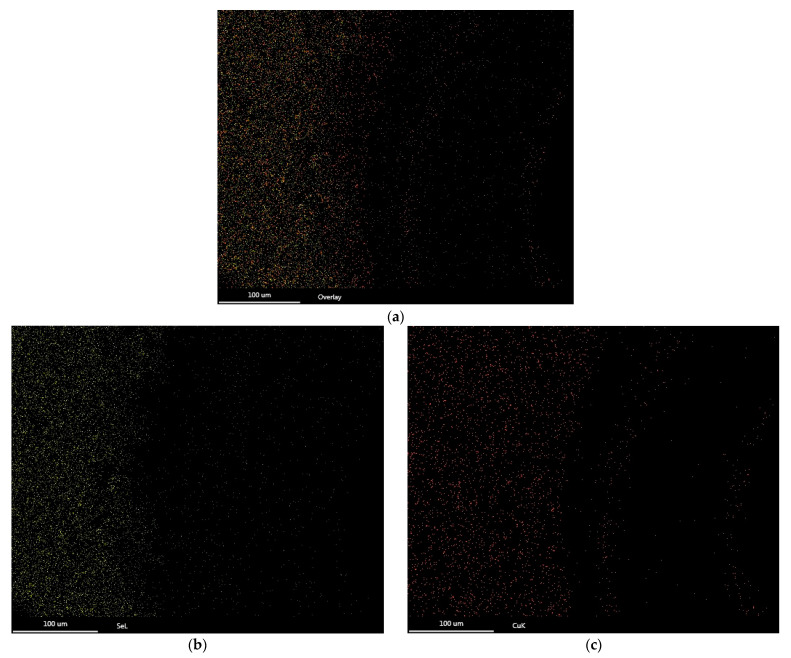
Elemental mapping of CuSe nanostructured coatings: (**a**) copper–selenium map, (**b**) selenium map, (**c**) copper map.

On the other hand, elemental mapping was carried out to determine the presence of copper and selenium nanoparticles in the coating. Figure 5a presents yellow (selenium) and red (copper) dots that indicate the homogeneous distribution of the elements present in the coating. The elemental mapping of each element presented in Figure 5b,c confirms the perfect distribution of selenium and copper particles. In the right area of the image, the presence of these points was not clearly observed because the substrate has a hole in this area. Both analyses were carried out in the same area of the substrate and it was observed that the number of copper particles present in the coating is lower than the number of selenium particles; this is in agreement with the percentages by weight presented in Table 1.

**Figure 6:**
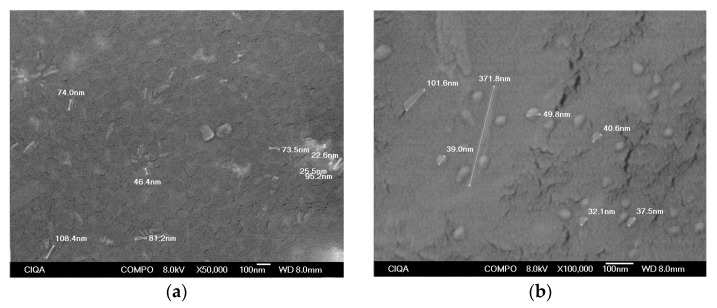
Scanning electron micrographs of CuSe nanostructured coatings at 3.0% wt. of CuSe nanoparticles. (**a**) Magnification at 50,000×, (**b**) magnification at 100,000×.

Scanning electron microscopy micrographs at 50,000× and 100,000× of the CuSe nanostructured coating at 3.0% wt. of CuSe nanoparticles are shown in Figure 6a and Figure 6b, respectively. In both magnifications, they show two types of morphologies: rod-shaped nanoparticles and spherical nanoparticles. The nanoparticles with spherical morphology have an average diameter in the range of 32.1 nm and 49.8 nm, while rod-shaped nanoparticles mainly presented longitudes of around 101.6 nm. Although both types of nanoparticles showed a tendency to form agglomerates, it was not possible to observe micron-sized agglomerates; the original spherical morphology was preserved even when the CuSe coating was subjected to heat treatment during deposition.

It has been reported that selenium and CuSe nanoparticles are structurally unstable and can change their size and morphology depending on the synthesis conditions, temperature, and composition, causing the formation of micrometric agglomerates [21,25], and the transformation can occur at low temperatures such as 25 °C [28]. This phenomenon was not observed in the coatings with CuSe nanoparticles even though they were subjected to temperatures of 130 °C during curing.

The results of the elemental analysis of the ion release test are shown in Table 2. These show that the nanoparticles were able to release ions through the polymer matrix and when the exposure time increases, the ion release also increases. A clear tendency can be seen in the release of ions, with selenium being released in a higher concentration in all cases, with respect to copper.

The phenomenon of ion release from nanoparticles embedded in a polymer matrix is achieved in several stages, but mainly three are involved in soluble matrices, which are the following:

1. The release by “burst”: This occurs when the ions that are closest to the surface are released; this could be due to a release due to the swelling of the material [39], to the bad interaction between the matrix and the active principle, or to the porosity of the matrix when it comes into contact with an environment that dissolves it [40].
2. The diffusional release: It is the stage where the ions begin to diffuse through the polyacrylate matrix [39,41].
3. Erosional release: This occurs when the material degrades due to environmental causes, which releases ions [39,41].

For systems with nanoparticles homogeneously distributed in the medium, only the last two stages are considered, while for those that are heterogeneously distributed, all three occur. For this case, it is considered that the nanoparticles are homogeneously distributed and settled on the coating surface.

Between days 40 and 80, the release of selenium ions decreased; this can be due to many factors as follows. First, the substrate could present limitations of ion release, where there could be erosion or diffusion through the membrane. Another explanation could be that at a time point between 20 and 80 days of exposure, there are impediments to the release of selenium ions because they are bonded as a compound with copper in addition to the fact that, as previously reported, the use of biopolymers as stabilizers in nanoparticles could slow down the release of these [42].

The results obtained of the release of ions from the coating show that the release occurs very slowly. This release rate suggests that there is a good interaction between the resin and the CuSe-GA nanoparticles, since if there were weak interactions between them, we would observe a sudden initial release [40,43], which is not optimal for applications such as coatings or paintings. These results are in agreement with the findings found in FTIR.

**Figure 7:**
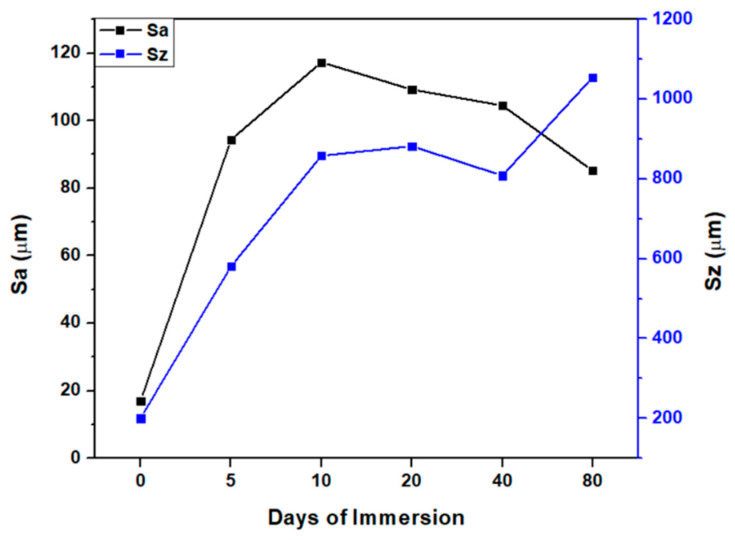
Surface roughness analysis of CuSe nanostructured coatings, arithmetic mean height (Sa), and maximum height (Sz).

Figure 7 presents the surface roughness of CuSe nanostructured coatings. The arithmetic mean height (Sa) starts with a value of 17.0 µm and increases to 117.3 µm after 10 days. From here, a decrease in the arithmetic mean is recorded, reaching 85.2 µm at 80 days.

On the other hand, the maximum height obtained (Sz) exhibits a behavior similar to the previous one, which confirms having the maximum height at 10 days and after that, there is a decrease in both coatings.

This observed behavior may be due to the interaction of water with the coating. In the first days of immersion, the coating tends to swell, which under the roughness analysis, indicates an increase in it.

After 10 days, the maximum peak of the roughness is observed, which would correspond to the maximum capacity of the coating to swell due to water, and from there, the coating begins to return to its original shape. The fact that the coating does not recover its original roughness indicates that the interaction with water generates an irreversible increase in roughness.

**Figure 8:**
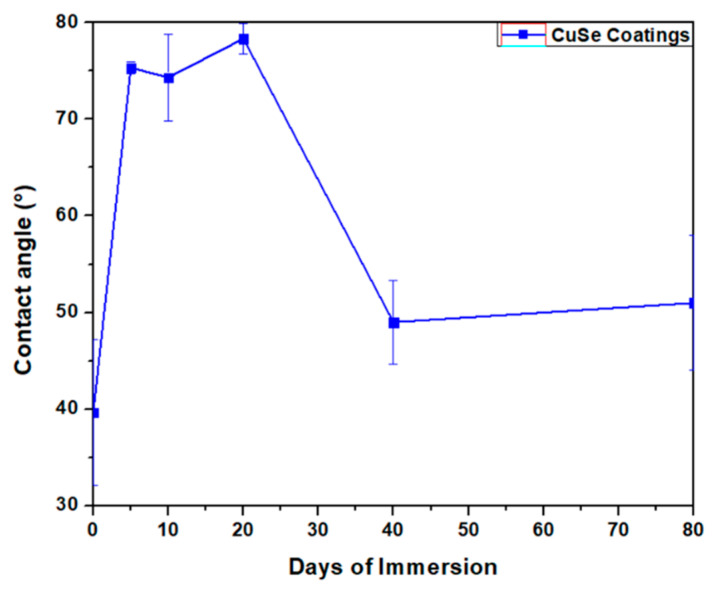
Contact angle measurements of CuSe nanostructured coatings.

Figure 8 presents the contact angle measurements of CuSe nanostructured coatings. The measurement of the contact angle of the CuSe nanostructured coating without water immersion (0 days) was 39.6 degrees. At 5, 10, and 20 days, different values of 75, 74, and 78 degrees were observed. These results indicate that the changes obtained in the surface of the nanostructured CuSe coating by immersion tend to exhibit a decrease in their hydrophilic behavior. However, at 40 and 80 days, there is a decrease in the contact angle of 49 and 51 degrees, respectively, indicating that the hydrophilic character increases after 20 days of immersion.

These coatings show a minor hydrophilic character between 5 and 20 days, indicating that immersion during the first days significantly alters their nature. The fact that this character is lost after 20 days suggests that the coating returns to a state similar to the initial one, and therefore that it recovers its hydrophilic character [44]. This experimental evidence explains the strong release of selenium and copper ions observed after 20 days.

### 3.2. Mechanical Tests Analysis

**Figure 9:**
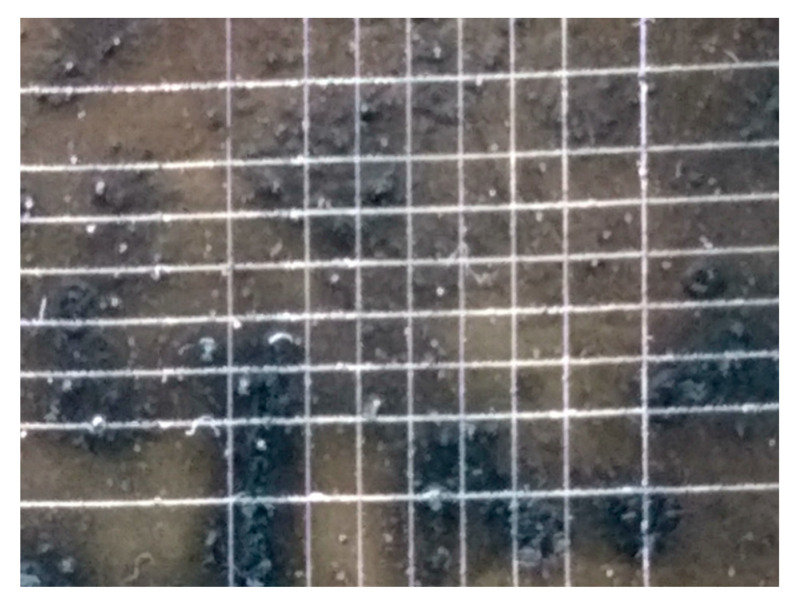
Mechanical performance tests (ASTM D3359) of CuSe nanostructured coating.

Figure 9 presents the results of the CuSe nanostructured coatings measured with an adhesion probe by a tape test. It can be appreciated that after the scratching and adhesion of the tape, the coating is maintained (having only detachment in the scratched area), which places it at a 5B rating, this being the highest. This indicates that the adhesion of these coatings is high, so it is expected that they do not come off easily under uncontrolled conditions.

We want to strongly emphasize that CuSe-coated steel substrates can be mechanically cut without peeling off the coating.

Although acrylic resin produces coatings with excellent mechanical resistance and allows the release of CuSe ions, the low values of the contact angle suggest that the adhesion of bacteria may be favored. Chemical surfaces that eliminate bacteria on contact require humidity from the medium, but this condition degrades the coating and its average lifetime decreases [29]. To extend the antimicrobial efficiency of CuSe coatings, it is necessary to apply a second layer of a superhydrophobic coating (θ ≥ 150°). This prevents the adhesion of bacteria by a physical mechanism and at the same time prevents the diffusion of moisture or contaminants in the CuSe coating layer. Once the superhydrophobic coating loses its hydrophobic property, the CuSe coating will be activated by releasing copper and selenium ions. Strategies that combine coatings with different mechanisms of action are attractive and present reduced environmental risks [29,30,31].

### 3.3. Microbiological Tests Results

**Figure 10:**
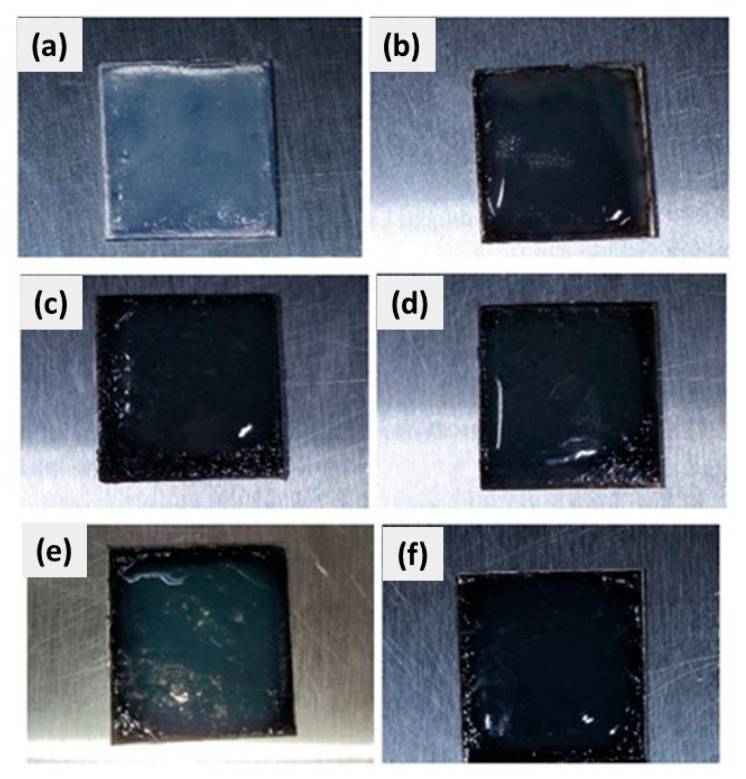
CuSe nanostructured coatings: (**a**) 0, (**b**) 0.5, (**c**) 1, (**d**) 1.5, (**e**) 2.0, and (**f**) 3.0% wt. with nutrient agar.

The results of microbiological analyses of CuSe nanostructured coatings are shown as follows. First, Figure 10 shows images of CuSe nanostructured coatings at different weight concentrations of CuSe nanoparticles covered with the culture medium (without inoculation). These coatings will serve as a blank control to compare with the inoculated coatings, for comparing color, texture, and possible microbial growth. The culture medium has a transparent, slightly yellow appearance. However, as the concentration of CuSe nanoparticles increases, the slightly yellow appearance becomes slightly darker.

**Figure 11:**
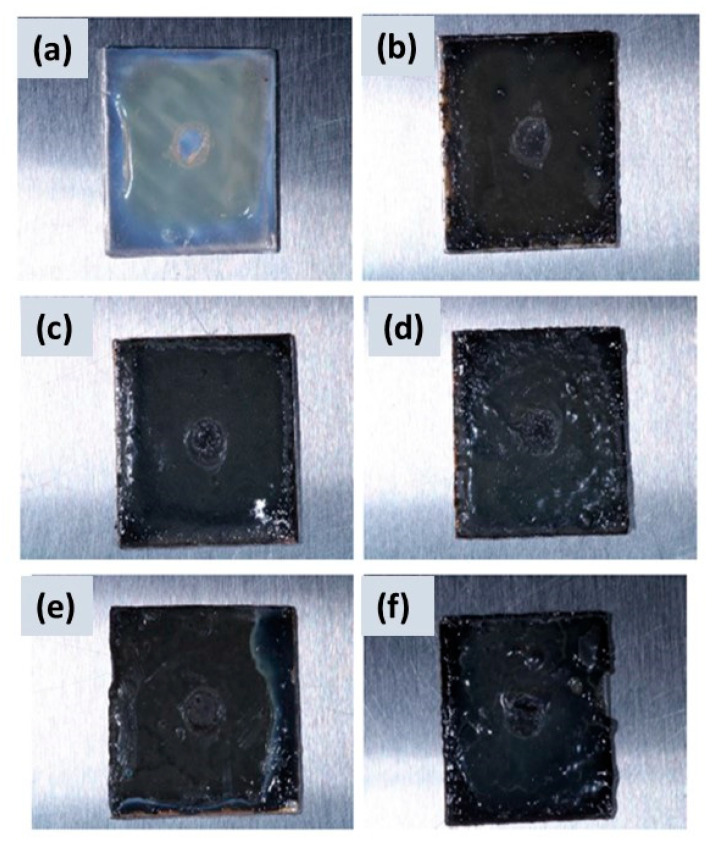
CuSe nanostructured coatings: (**a**) 0, (**b**) 0.5, (**c**) 1.0, (**d**) 1.5, (**e**) 2.0, and (**f**) 3.0% wt. inoculated with *Escherichia coli*.

**Figure 12:**
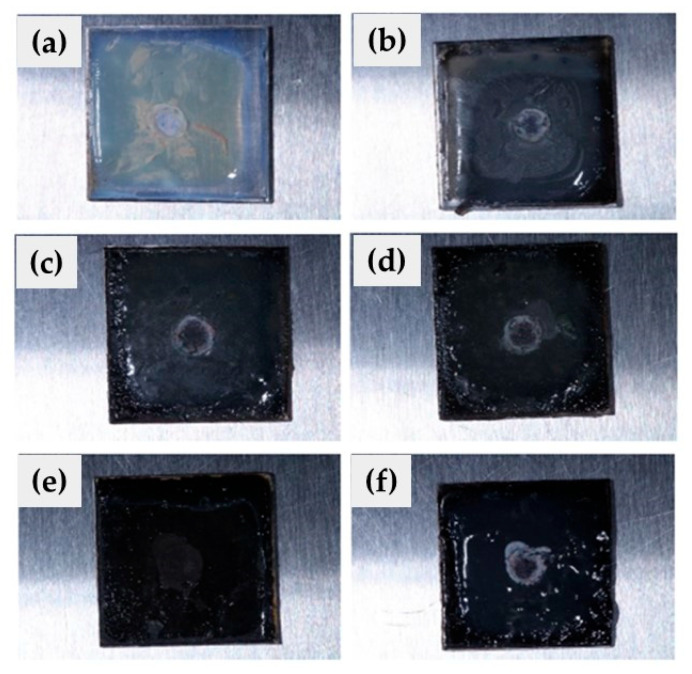
CuSe nanostructured coatings: (**a**) 0, (**b**) 0.5, (**c**) 1.0, (**d**) 1.5, (**e**) 2.0, and (**f**) 3.0% wt. inoculated with *Candida albicans*.

Figure 11 and Figure 12 exhibit images of the microbial growth of both *E. coli* and *C. albicans* strains in contact with nanostructured coatings at different CuSe concentrations.

In Figure 11, we can observe images that show the CuSe nanostructured coatings inoculated with *Escherichia coli*. Comparing Figure 11a and Figure 10a, it can be seen that Figure 11a shows a yellow coloration and a milky white halo around the inoculation zone; this suggests that microbial growth spread throughout the culture medium due to the moist conditions present on the surface. In Figure 11b–f, the substrates did not present a yellow coloration on the entire surface and only maintained the white halo in the incubation zone. This again suggests microbial growth only in the incubation zone (possibly due to the removal of the CuSe coating), and the proliferation of *Escherichia coli* bacteria through the culture medium was not favorable due to the release of copper and selenium ions. These results suggest that CuSe nanostructured coatings in concentrations of 0.5 to 3.0% by weight of CuSe nanoparticles have an inhibitory effect on the growth of *E. coli* on nutrient agar.

Figure 12 presents nanostructured CuSe coatings inoculated with *Candida albicans* studied under the same conditions as the bacteria *Escherichia coli.* The substrate without CuSe NPs, illustrated in Figure 12a, presented characteristics similar to those observed in Figure 11a, that is, a yellow coloration and a white halo at the inoculation site. The substrates with CuSe NPs (Figure 12b–f) also presented a white halo with greater intensity that indicates microbial growth exclusively in the inoculation zone.

The CuSe nanostructured coatings were cleaned with 70% ethanol and washed with water to determine the number of colony-forming units (CFUs), following a previous report [34]. In the plates seeded from the dilutions, a decrease in the logarithmic number was evident with the presence of the growth of bacteria placed in contact with coatings compared to the control.

The antimicrobial activity studies illustrated in Figure 11 and Figure 12 suggest that CuSe coatings have hydrophilic characteristics (contact angles less than 90°) that favor the dispersion of moisture on the surface and the release of copper and selenium ions preventing the proliferation of *Escherichia coli* and *Candida albicans*.

**Figure 13:**
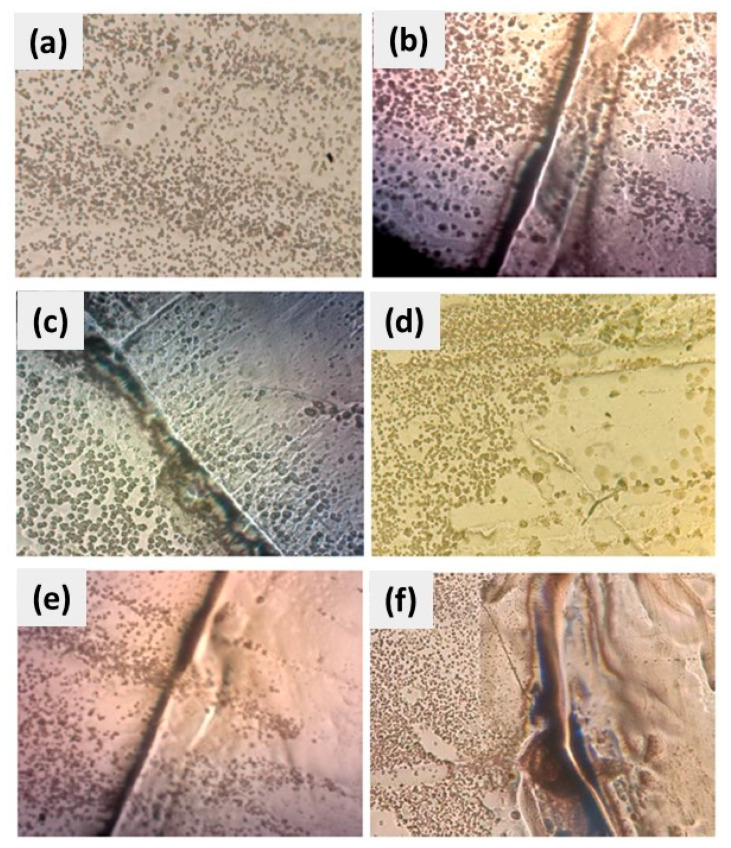
Images of the inhibition evaluation of the direct contact of a CuSe nanostructured coating: (**a**) 0, (**b**) 0.5, (**c**) 1.0, (**d**) 1.5, (**e**) 2.0, and (**f**) 3.0% wt. with *Escherichia coli* strain.

**Figure 14:**
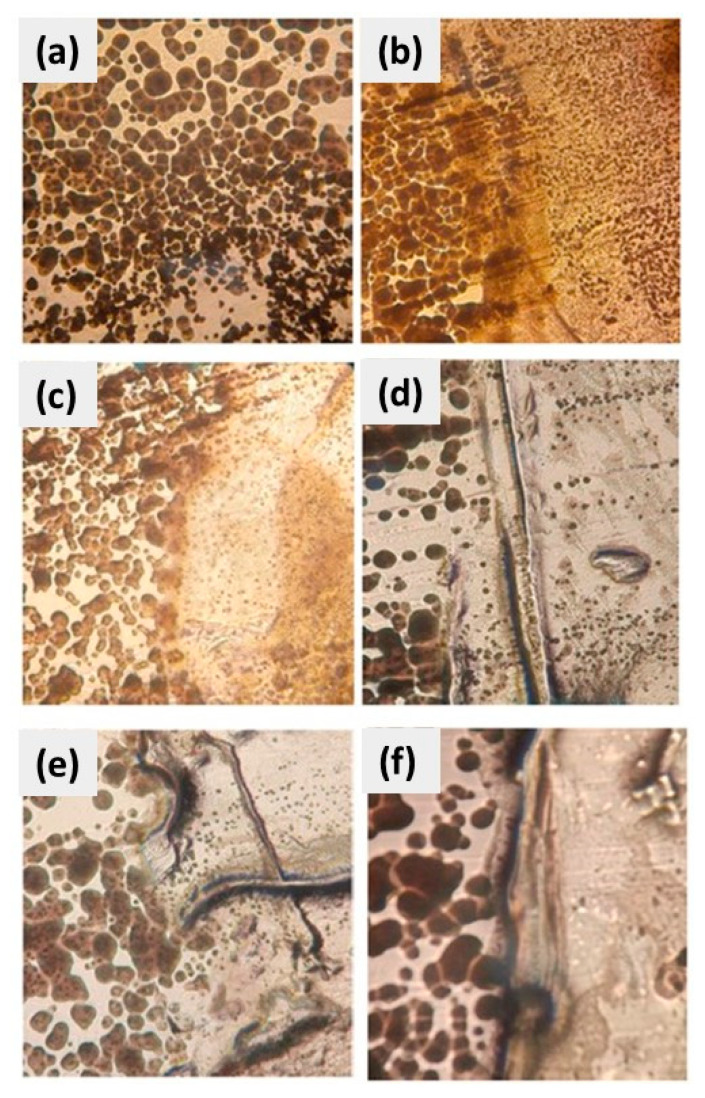
Inhibition evaluation of the direct contact of a CuSe nanostructured coating: (**a**) 0, (**b**) 0.5, (**c**) 1.0, (**d**) 1.5, (**e**) 2.0, and (**f**) 3.0% wt. with *Candida albicans*.

After biological contamination, Figure 11 and Figure 12, the substrates were cleaned with ethanol and water. The CuSe nanostructured coatings were then removed in the form of a film from the metal substrates by scraping and placed directly on Petri dishes with nutrient agar inoculated with *Escherichia coli* and *Candida albicans* (Figure 13 and Figure 14, respectively). The concentrations of CuSe nanostructured coatings employed were the same as in the previous experiment, 0, 0.5, 1.0, 1.5, 2.0, and 3.0% wt. of CuSe nanoparticles. Figure 13 and Figure 14 show the presence of two zones separated by a central line, the zone located on the left corresponds to microbial growth under optimal conditions and the zone on the right corresponds to microbial growth in the presence of the nanostructured CuSe coating.

Figure 13 shows the images of CuSe nanostructured coatings at different concentrations at wt.% in direct contact with the *E. coli* strain. Figure 13a represents a blank control where no CuSe nanostructured coating was used.

On the side exposed to the CuSe nanostructured coatings, microbial growth is lower than on the opposite side. This inhibition increases as CuSe nanoparticles’ concentration increases, as seen in the image of CuSe 2.0 wt. % and higher concentrations. These results match with the previous experiment, where the microbial growth inhibition caused by CuSe nanostructured coatings for *Escherichia coli* was demonstrated.

Figure 14 presents images of the inhibition evaluation of the direct contact of a CuSe nanostructured coating with the *Candida albicans* strain. Figure 14a shows the image of the blank where no CuSe nanostructured coating was used.

As in the counterpart with *E. coli*, the inhibition produced by the CuSe nanostructured coatings can be appreciated. This inhibition is observed clearly in all the treatments and becomes clearer as the CuSe concentration increases. These results confirm that the CuSe nanostructured coatings have an inhibitory effect, in direct contact, on microbial growth in *Candida albicans*.

The results of antimicrobial activity against *Escherichia coli* and *Candida albicans* indicated that the CuSe NPs are distributed over the entire surface of the coating (confirmed by SEM studies) and it is likely that a good number of NPs settle at the bottom of the coating during the application and curing process of the coating [39,40,41]. The use of CuSe biocides in antifouling coatings could be an excellent alternative due to the environmental regulations imposed on copper-based coatings, but it will be necessary to carry out toxicity studies to know the potential of these materials [45].

## 4. Conclusions

CuSe nanostructured coatings were successfully obtained employing previously synthesized CuSe nanoparticles modified with gum arabic, embedded in a commercial resin.

Despite the surface inhomogeneity of the coatings, the scratch adhesion tests showed great adhesion, in part due to the interaction present among the CuSe nanoparticles, the gum arabic, and the commercial resin determined by FTIR.

Elemental analysis using EDS shows that the nanostructured coating is composed mainly of oxygen and carbon, elements that belong to the resin and gum arabic. Besides these elements, selenium and copper appear. Selenium is in a higher proportion than copper. On the other hand, elemental mapping shows that both selenium and copper are well dispersed in the coating, with selenium found in a higher proportion.

The micrographs obtained by electron microscopy show that the CuSe nanostructured coatings are composed of some agglomerates and mainly of spherical particles with an average particle diameter in the range of 32.1 to 49.8 nm.

The nanostructured CuSe coating was shown to have excellent adhesion (ASTM D3359 standard), with a rating of 5B, which is the highest (having only detachment in the scratched area). This result indicates that the mechanical properties of the coating are outstanding, so it would be expected to be very resistant and not easily detached under hostile environmental conditions.

The release of selenium and copper ions in these coatings was verified, where selenium ions were released in greater proportion. During the drying and curing of the coating, the CuSe NPs remain embedded in the resin matrix; this process leads to the good mechanical resistance of the coating and favors the slow release of the ions, which are responsible for the inhibition of the microbial growth of the microorganisms studied, *Escherichia coli* and *Candida albicans*.

## Figures and Tables

**Table 1:** Weight percent of element concentrations in CuSe nanostructured coatings.

Element	Wt. %
Oxygen	77.4
Carbon	20.62
Selenium	1.14
Copper	0.84

**Table 2:** Ion release tests elemental analysis.

Sample Supernatant	Se (ppm)	Cu (ppm)
Blank (distilled water)	0	0
Black 5 days	0.088	0.009
Black 10 days	0.125	0.014
Black 20 days	0.414	0.034
Black 40 days	0.737	0.047
Black 80 days	0.519	0.132

## Data Availability

The data presented in this study are available on request from the corresponding authors.
